# Editorial: COVID-19 in CNS and PNS: Basic and Clinical Focus on the Mechanisms of Infection and New Tools for the Therapeutic Approach

**DOI:** 10.3389/fneur.2022.838227

**Published:** 2022-03-03

**Authors:** Jorge Matias-Guiu, Jordi A. Matias-Guiu, Carmen Garrido, Genaro Pimienta, Patricio F. Reyes, Abdul Mannan Baig, Ulises Gomez-Pinedo

**Affiliations:** ^1^Department of Neurology, Institute of Neurosciences, Instituto de Investigación Sanitaria del Hospital Clínico San Carlos (IdISSC), Hospital Clínico San Carlos, Universidad Complutense de Madrid, Madrid, Spain; ^2^Laboratory of Neurobiology, Institute of Neurosciences, Instituto de Investigación Sanitaria del Hospital Clínico San Carlos (IdISSC), Hospital Clínico San Carlos, Universidad Complutense de Madrid, Madrid, Spain; ^3^Institut National de la Santé et de la Recherche Médicale (INSERM), Paris, France; ^4^Sanford Burnham Prebys Medical Discovery Institute, La Jolla, CA, United States; ^5^Barrow Neurological Institute (BNI), Phoenix, AZ, United States; ^6^Department of Biological and Biomedical Sciences, Aga Khan University, Karachi, Pakistan

**Keywords:** COVID 19, central nervous system, SARS-CoV2, neurological diseases, persistent COVID

The COVID-19 pandemic has had significant implications not only for health but also for lifestyles and for organizations both in the healthcare sector and in other spheres. Before December 2019, other coronaviruses had been described that had presented endemically and in some cases caused epidemics, but none spread to such an extent globally; the COVID-19 pandemic is reminiscent of other historic epidemics such as the influenza epidemic of the last century, whose sequelae, including neurological symptoms, were felt for decades (1). Of the other coronaviruses (CoV) affecting humans, 2 are classified as αCoV (HCoV-229E and HKU-NL63) and 4 as βCoV (HCoV-OC43, HCoV-HKU1, SARS-CoV, and MERS-CoV). SARS-CoV and MERS-CoV caused severe infections both in healthy individuals and in people with immune deficiencies, and were associated with high mortality rates, whereas the others are associated with seasonal outbreaks of flu-like symptoms with relatively limited capacity for transmission. However, all of these viruses have either been associated with neurological symptoms or been detected in biological samples taken from the central nervous system (CNS) (2, 3). On 31 December 2019, the World Health Organization reported a novel CoV (SARS-CoV-2) in patients with pneumonia in the city of Wuhan, in the Chinese province of Hubei, which spread rapidly through the rest of the world. The novel virus is a βCoV and bears considerable similarity to SARS-CoV. The main structural differences between SARS-CoV and SARS-CoV-2 are observed in the fusion protein and in accessory proteins, particularly ORF3b and ORF8. As is the case with SARS-CoV, subunit 1 of the novel coronavirus fusion protein also binds to the ACE2 receptor, hence the name SARS-CoV-2 (4). It has been suggested since early in the pandemic that the virus may have direct and indirect effects on the CNS (5), with many researchers and authors beginning to analyze a pandemic with worldwide effects; an enormous amount of information has been generated, largely due to the scientific community's desire for progress. However, much of this information is from case reports, anecdotal series, and many speculative articles based more on opinion than experimentation (6). Despite the great efforts to generate evidence to enable advances in our understanding of the virus and associated disease, much speculation remains today with regard to the evidence. Such aspects as its epidemiology, transmission factors, and the impact of mass vaccination are conditioned by data from studies that present considerable bias (7) and are not evidence-based.

Since the beginning of the pandemic, there has been debate as to whether SARS-CoV-2 is able to enter the CNS and cause neurological symptoms, trigger or promote pre-existing neurological disease, or remain latent in the brain, making the organ a viral reservoir (5). The study of the potential routes by which the virus enters the CNS (i.e., the hematogenous route or the neuronal route), facilitating this penetration, also constitute an interesting area of research (8, 9) analyzing whether the virus accesses the CNS by infecting endothelial or epithelial cells of the blood-brain barrier; by dissemination from nearby areas; or by axonal transport after infecting neurons in the peripheral nervous system. Another subject of debate is the frequency of neurological symptoms associated with the acute infection (10, 11) and their association with the CNS (12), and associated neurological disorders such as stroke (13, 14) and neuromuscular involvement (15). Furthermore, there is an interesting debate as to whether the virus can trigger neurological disease, for example neurodegenerative disease, in the long term (it has been suggested that the infection may increase the risk of Parkinson's disease or Alzheimer's disease), and by which mechanisms (16, 17), and whether it may facilitate age-related transcriptome or molecular changes (18). Finally, there is controversy regarding the so-called persistent COVID-19 or post–COVID-19 syndrome, referring to symptoms persisting for 3 months after the acute infection, with particular emphasis on cognitive symptoms (19). A recent Delphi consensus study conducted by the World Health Organization establishes 3 major criteria for diagnosing post–COVID-19 condition, 2 of which are based on the presence of neurological symptoms: fatigue and cognitive alterations (20).

The special issue “COVID-19 in CNS and PNS: Basic and Clinical Focus on the Mechanism of Infection and New Tools for the Therapeutic Approach” includes valuable articles addressing these debates, contributing evidence to expand our understanding of the disease and its impact on the CNS.

## Contributions on Neurological Symptoms During the Acute Phase

Several articles have contributed new information on neurological manifestations of COVID-19. For instance, Yan et al. analyze neurological symptoms in a retrospective series of 1,682 patients from Wuhan, with 30.3% presenting neurological symptoms (12.8% with headache). Tsai et al. reviewed 79 studies, selecting 63 for inclusion in a meta-analysis. These researchers report olfactory alterations in 35% of patients, headache in 10.7%, and stroke in 8.1%. Kushwaha et al. conducted a cross-sectional study analyzing data from 358 patients with neurological symptoms, 69 of whom had suspected SARS-CoV-2 infection, and evaluate the impact of the infection at a center primarily attending neurological patients. Pinzon et al. reviewed 280 studies, selecting 33 for meta-analysis, and report neurological alterations such as myalgia in 19.2% of patients, headache in 10.9%, and stroke in 4.4%; they also review the frequency and characteristics of neurological symptoms during the pandemic. Fiani et al. present an extensive review addressing the impact of neurological manifestations and the underlying mechanisms. The article by Gori et al. specifically studies the frequency and pathogenesis of anosmia, taking a very broad approach, analyzing the possible pathogenic mechanisms and addressing the current controversies; Mathew also contributes an interesting article on anosmia. Hwang et al. analyzed the presence of seizures during SARS-Cov-2 infection in 4 of their own patients and review the literature on the subject, whereas Waters et al. describe the incidence of electroencephalographic seizures. Zito et al. analyzed Guillain-Barré syndrome in patients with COVID-19 in a case report and meta-analysis of 29 articles. Jungbauer et al. analyze vocal cord palsy associated with SARS-CoV-2 infection. The study by Varela Rodríguez et al. addresses neuropsychiatric symptoms in patients with COVID-19 and history of alcohol abuse. Román et al. present a comprehensive clinical review of transverse myelitis associated with COVID-19 and vaccination against the disease, a highly informative contribution. Severa et al. discuss treatment with interferon beta in the context of COVID-19. Together, these articles offer a panoramic view of the neurological symptoms associated with COVID-19.

## Contributions on the Mechanism of Central Nervous System Invasion

This special issue includes several key articles. Huang et al. review the association between biological and molecular factors associated with the viral infection and their potential effects on the CNS. They propose the murine hepatitis virus as a model for studying the role of coronaviruses in the CNS; Sanclemente-Alaman et al. also highlight this model in their review of experimental models of SARS-CoV-2. Wang et al. review the action mechanisms of viruses affecting the CNS in an article on neurological symptoms of COVID-19 and offering an overview of the clinical manifestation and infection mechanisms. Reza-Zaldívar et al. analyze specific mechanisms associated with SARS-CoV-2. Gomes de Assis et al. address the underlying mechanisms of neurological symptoms associated with COVID-19 after reviewing 484 articles according to a pre-established methodology addressing different fields, such as host factors, immune mechanisms, and virology. This comprehensive study presents both experimental and clinical data. Guadarrama-Ortiz et al. conducted an exhaustive review of neurological manifestations, viral entry routes, and potential immune and virological mechanisms, discussing a broad range of potential neurological alterations affecting both the central and the peripheral nervous systems. This interesting review presents an overview of the relationship between SARS-CoV-2 and neurological symptoms. The virus is very difficult to detect or undetectable in the cerebrospinal fluid of patients with SARS-CoV-2 infection, even in those with encephalitis, according to the transcriptome analysis of cerebrospinal fluid samples by Placantonakis et al.; Pacheco-Herrero et al. studied neuropathological aspects of the infection.

## Contributions on Host Factors and the Risk of Neurological Disease

Bhaskar et al. comprehensively review the immune response associated with SARS-CoV-2 infection, placing special emphasis on the cytokine storm, as well as immunosenescence, and the role of these mechanisms in complications. Severa et al. analyzed the possible relationship between the use of interferon beta and the risk of infection, as well as the drug's role in the clinical course of the disease, considering its potential therapeutic role in COVID-19, as occurred in previous coronavirus epidemics.

## Contributions on Neurological Sequelae After Acute SARS-COV-2 Infection and Persistent COVID Syndrome

D'Arcy et al. discuss the long-term consequences of the infection. Fiani et al. analyzed persistent neurological symptoms after the infection, calling attention to the need for guidelines addressing sequelae of stroke, intracranial infections, and muscle damage, as well as nutrition-related issues.

## Moving Toward a Better Understanding of the Effects of SARS-COV-2 on the CNS and their Long-Term Implications

It remains to be determined whether the virus or its RNA can remain within structures of the CNS or whether it may remain latent or cause disease in the long term; therefore, there is a clear need for necropsy studies (21, 22). It also seems important to identify any areas of the CNS that may present greater vulnerability to infection (though the hippocampus and basal ganglia have been suggested), whether some CNS cells present greater susceptibility to viral structures, and whether the virus or its RNA may be transported by such cell substructures as vesicles or exosomes. It is also unclear whether existing lesions, such as demyelinating plaques in multiple sclerosis, may facilitate the entry of the virus and serve as viral reservoirs. The impact of different types of vaccine on viral invasion of the CNS is another area where further study is needed. The effects of SARS-CoV-2 on the brain constitute a new challenge for neuroscientific research (23), in which we may only advance with the greatest possible quantity of information, like that included in this special issue.

## Author Contributions

JM-G, UG-P, JAM-G, CG, GP, PR, and AB concept, evaluation, writing, and revision. All authors contributed to the article and approved the submitted version.

## Conflict of Interest

The authors declare that the research was conducted in the absence of any commercial or financial relationships that could be construed as a potential conflict of interest.

## Publisher's Note

All claims expressed in this article are solely those of the authors and do not necessarily represent those of their affiliated organizations, or those of the publisher, the editors and the reviewers. Any product that may be evaluated in this article, or claim that may be made by its manufacturer, is not guaranteed or endorsed by the publisher.
